# Supplementary material for: Gender disparities in social and personality psychology awards from 1968 to 2021
Source: Commun Psychol. 2024 Jul 3;2:63. doi: 10.1038/s44271-024-00113-5 (PMC11332211; doi:10.1038/s44271-024-00113-5)
Supplement: Supplementary file 2 — Supplementary information file (pdf) [file 44271_2024_113_MOESM2_ESM.pdf]

Supplementary Information for:

Gender Disparities in Social and Personality Psychology Awards from 1968 to 2021

This file includes:

Supplementary Table 1

Supplementary Figure 1

Supplementary Figures 2-5

Supplementary Figure 6-7

Supplementary Table 1.

Supplementary Table 1. Logistic regression analysis for variables predicting woman (vs. man) award recipient controlling for clustering within society.

Predictor	OR	SE	z	95% CI OR Lower, Upper		*p*
(intercept)	0.38	0.05	-6.88	0.29	0.50	< .001
Year	1.03	0.01	2.84	1.01	1.05	.004
Award Type: reference Research						
Impact/Media	1.38	0.34	1.30	0.85	2.24	.195
Service	2.12	0.41	3.92	1.45	3.08	< .001
Teaching	1.63	0.41	1.93	0.99	2.66	.054
Award Level: reference Senior						
Mid-Career	0.65	0.30	-0.93	0.27	1.61	.354
PG/ECR	2.27	0.31	6.08	1.74	2.96	< .001
All levels	1.43	0.23	2.25	1.05	1.95	.024
Shared	1.01	0.12	0.13	0.81	1.28	.900
Hon. Mention	1.10	0.18	0.56	0.80	1.50	.574
Year x Impact/Media	1.00	0.02	0.02	0.96	1.04	.986
Year x Service	1.05	0.02	2.22	1.01	1.09	.027
Year x Teaching	1.08	0.04	2.34	1.01	1.16	.019
Year x Mid-Career	1.10	0.07	1.51	0.97	1.24	.132
Year x PG/ECR	0.99	0.01	-0.56	0.96	1.02	.579
Year x All levels	0.99	0.01	-0.87	0.95	1.02	.382
Year x Shared	1.00	0.01	0.06	0.98	1.02	.955
Year x Hon. Mention	1.00	0.01	0.03	0.97	1.03	.973
Random Effects						
σ^2	3.29					
τ_{00} Society	0.09					
ICC	0.03					
N Society	17					
Observations	2629					
Marginal R^2	0.080					
Conditional R^2	0.103					

Note. Hon. = Honorable. Year is mean centered.

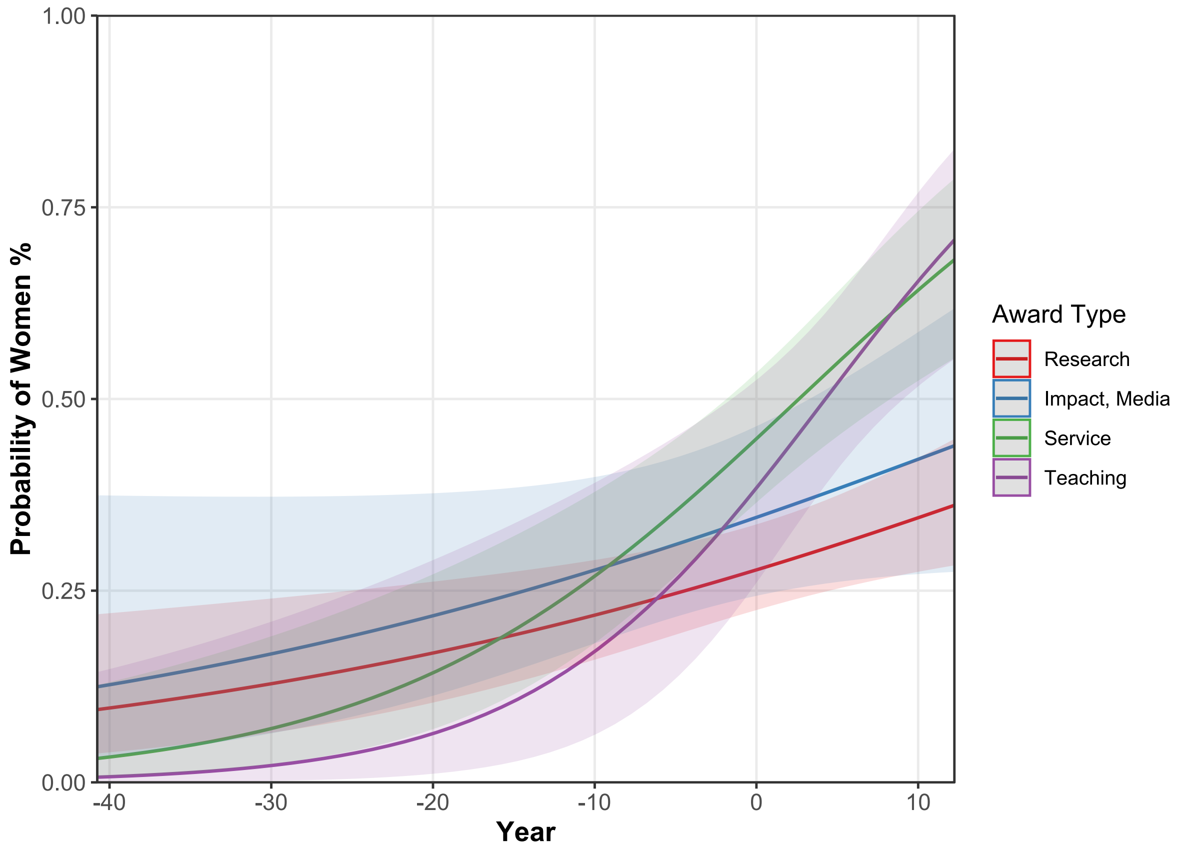

Supplementary Figure 1. Probability of award recipient being a woman by award type over time controlling for clustering within society. Line represents the slope, and colored area represents the 95% confidence intervals. Red indicates research awards; blue indicates impact and media awards; green indicates service awards; purple indicates teaching awards. Year is mean centered.

Supplementary Figures 2-5

As shown in Supplementary Figure 2, men outstripped women in research and impact/media awards over time, although women have increased their share of research awards over time. In contrast, for service women's proportions of awards have increased steadily over time and outstripped men's in 2016-2021 for the first time. Whereas for teaching women's share of awards has kept pace with men's or outstripped them but only since 2001-2005 and 2011-2015, respectively.

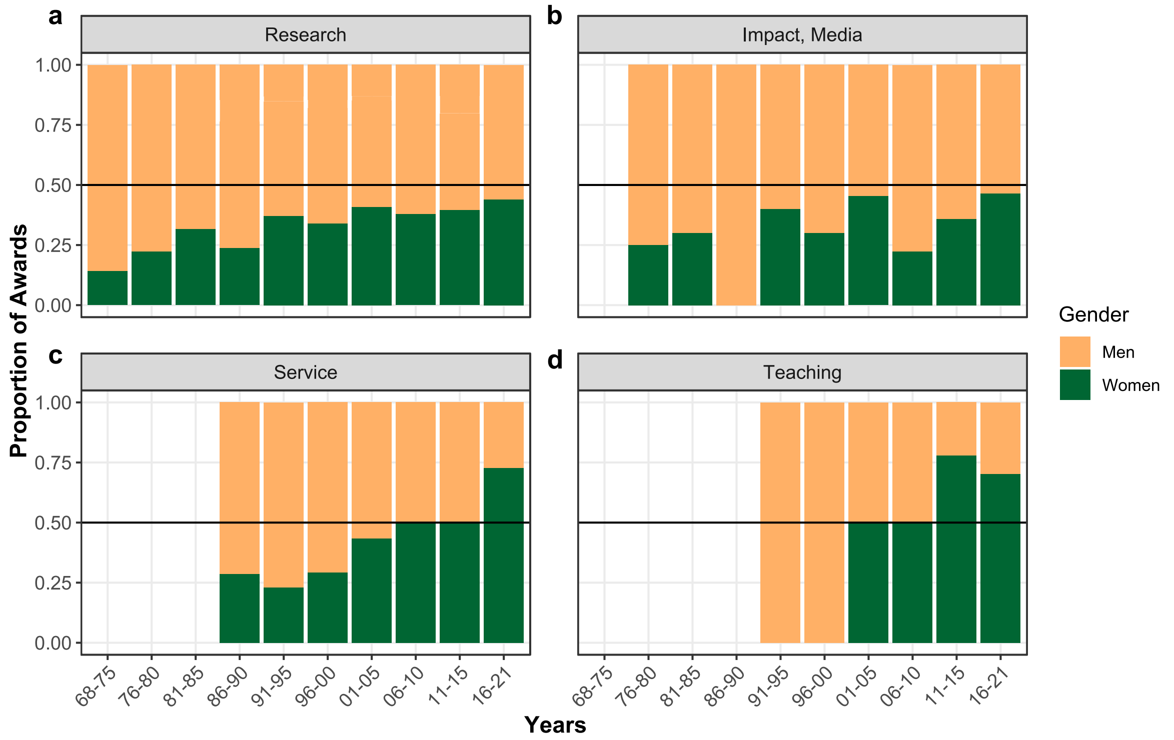

Supplementary Figure 2. Proportion of awards given to women and men across time at five year intervals by award type. All available data included except winners categorized as not applicable for gender/sex. Green indicates women; yellow indicates men. **a:** research awards; **b:** impact and media awards; **c:** service awards; **d:** teaching awards.

As shown in Supplementary Figure 3, a similar pattern was found over time for award level: men took home more awards at senior and mid-career, but women's share of awards at these levels has increased overtime, almost reaching gender parity for senior awards between 2016-2021. For PG/ECR, the proportion of awards going to men and women was roughly

equal across years. For all levels, women's share of awards has increased over time and remains close to 50% since 2011.

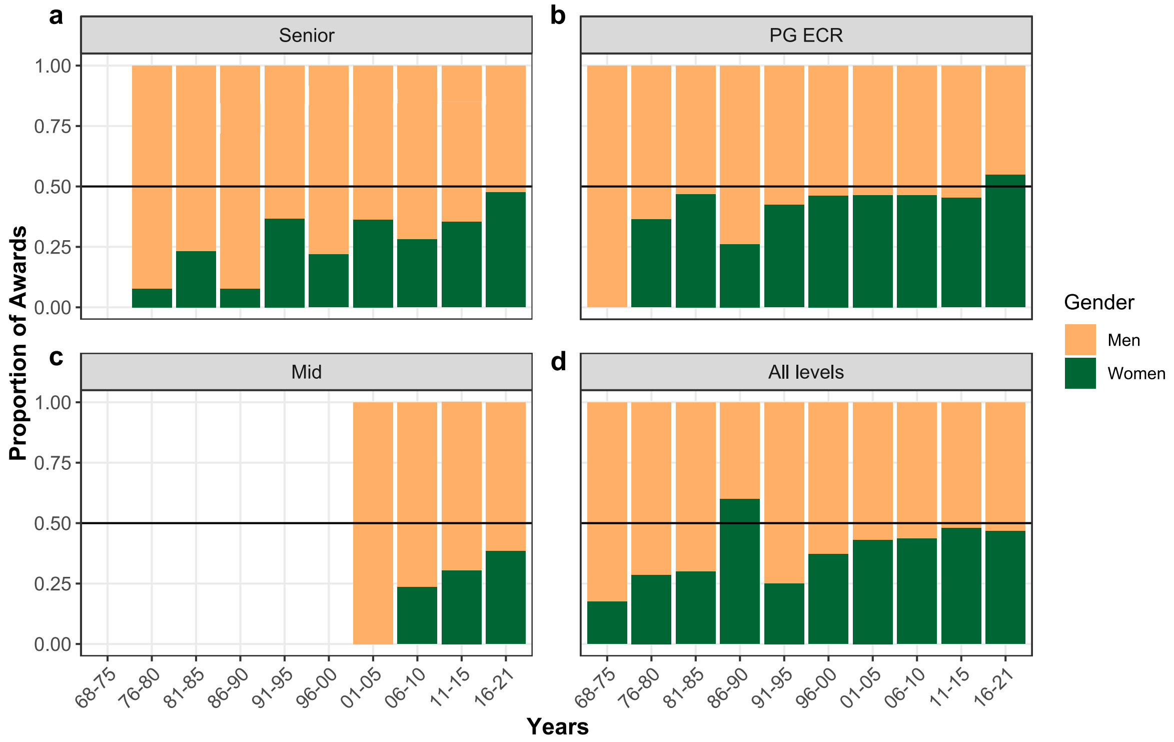

Supplementary Figure 3. Proportion of awards given to women and men across time at five year intervals by award level. All available data included except winners categorized as not applicable for gender/sex. PG = Postgraduate; ECR = Early Career Researcher. Green indicates women; yellow indicates men. **a:** senior level; **b:** PG/ECR level; **c:** mid-career level; **d:** all levels.

As shown in Supplementary Figure 4, over time there was a general trend towards gender/sex parity for awards categorized as shared and not shared.

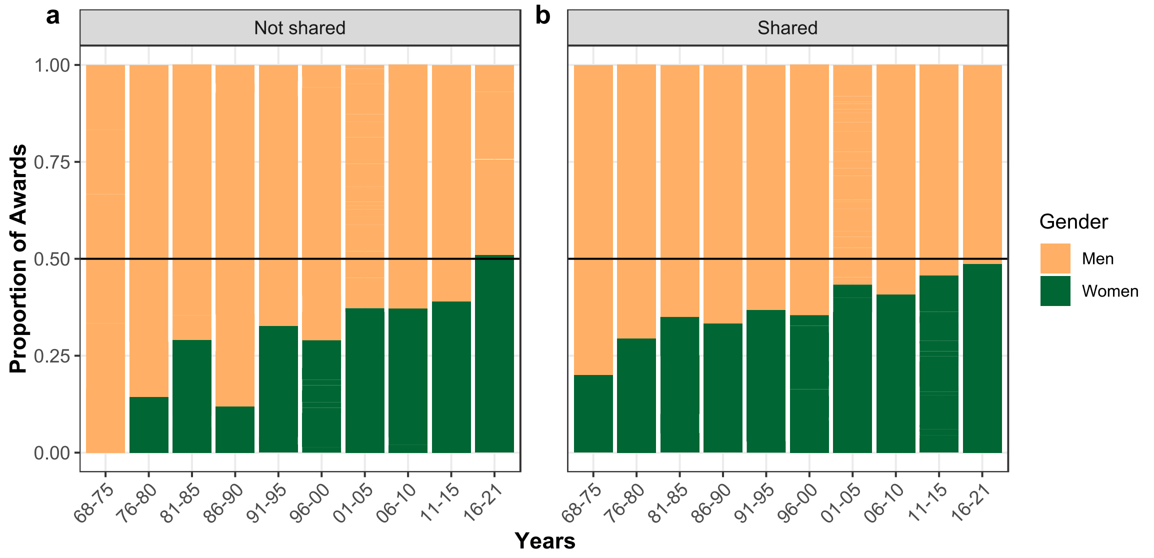

Supplementary Figure 4. Proportion of shared and not shared awards given to women and men across time at five year intervals. All available data included except winners categorized as not applicable for gender/sex. Green indicates women; yellow indicates men. **a:** not shared awards; **b:** shared awards.

As seen in Supplementary Figure 5, men received more honorable mentions in earlier years, except between 1981-1985 when women received a greater proportion of honorable mentions than men. Since then, women have steadily increased their proportion of honorable mentions, with women outstripping men since 2011. In contrast, when the award did not include honorable mentions, men outstripped women in proportion of awards received, albeit there was greater gender/sex parity in later years.

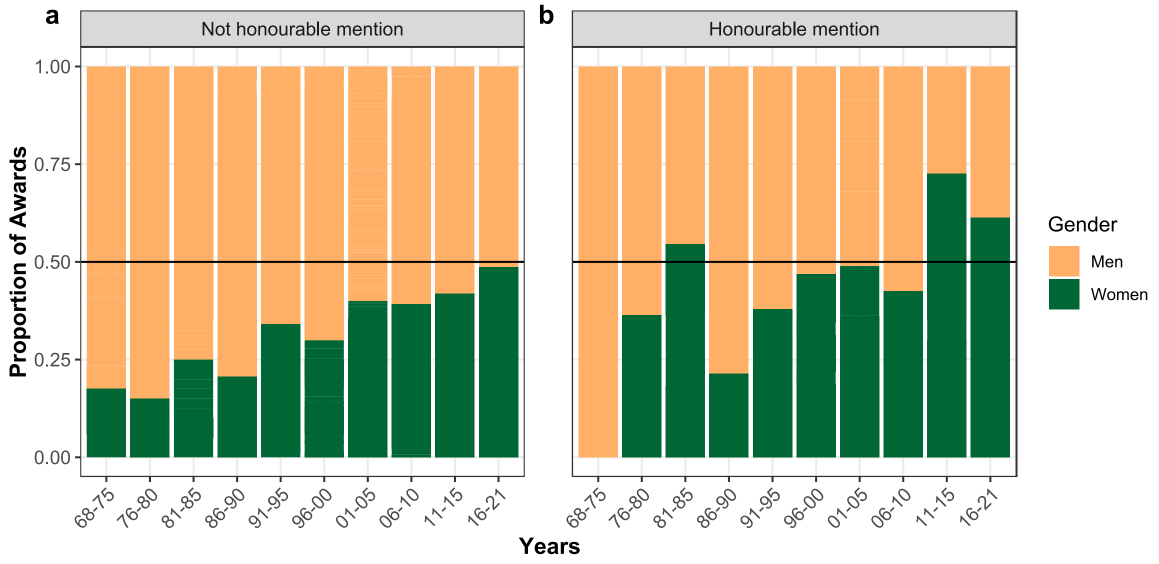

Supplementary Figure 5. Proportion of honorable mentions and not honorable mentions given to women and men across time at five year intervals. All available data included except winners categorized as not applicable for gender/sex. Green indicates women; yellow indicates men. **a:** not honorable mentions; **b:** honorable mentions.

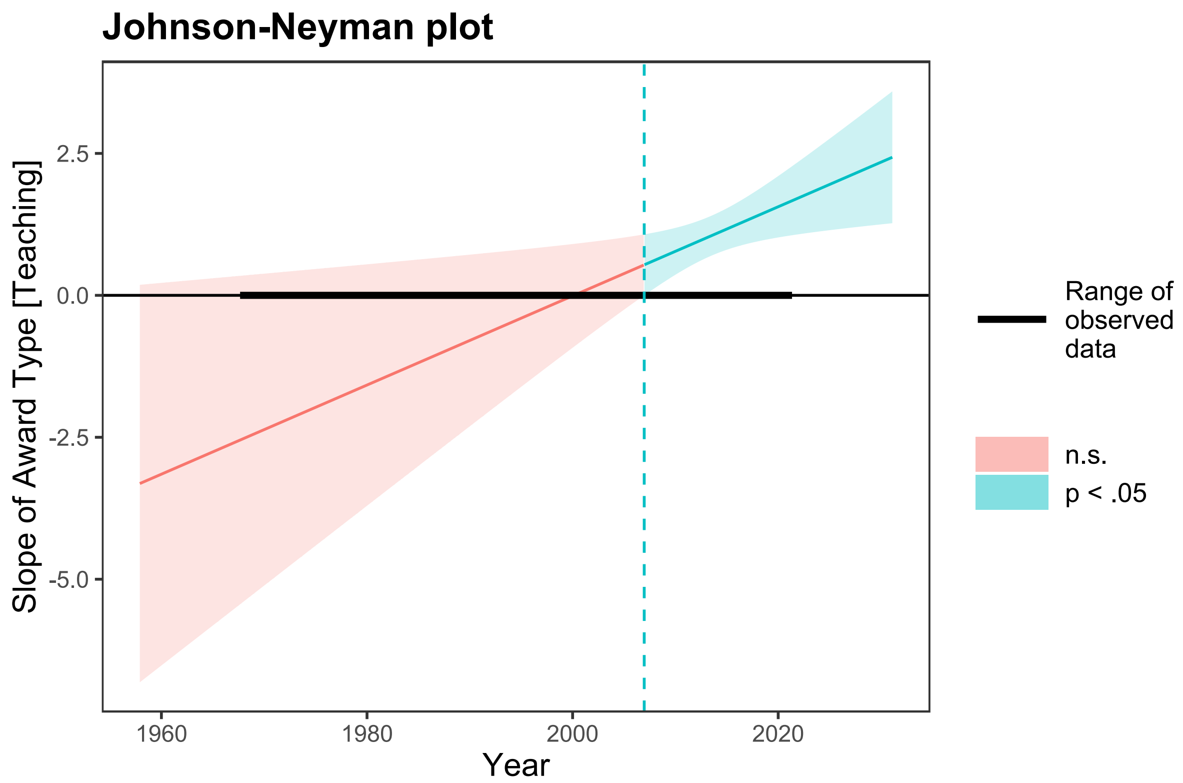

Supplementary Figure 6. Johnson Neyman plot for award type (teaching) by year interaction. Range of observed data for year is 1968 to 2021. Dashed line represents the point at which the slope for award type (teaching) becomes significant at $p < .05$. Colored areas represent 95% confidence intervals. Red indicates non-significant; blue indicates significant at $p < .05$.

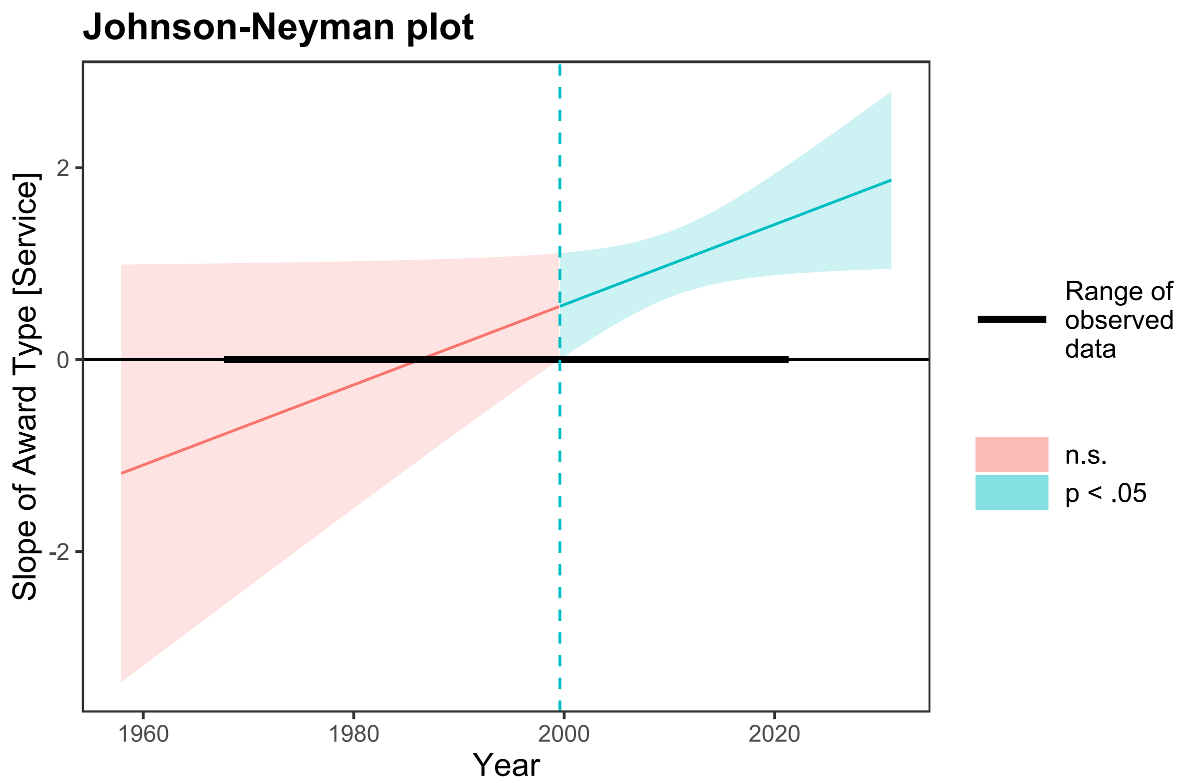

Supplementary Figure 7. Johnson Neyman plot for award type (service) by year interaction. Range of observed data for year is 1968 to 2021. Dashed line represents the point at which the slope for award type (service) becomes significant at $p < .05$. Colored areas represent 95% confidence intervals. Red indicates non-significant; blue indicates significant at $p < .05$.
