# Supplementary material for: Gender disparities in social and personality psychology awards from 1968 to 2021
Source: Commun Psychol. 2024 Jul 3;2:63. doi: 10.1038/s44271-024-00113-5 (PMC11332211; doi:10.1038/s44271-024-00113-5)
Supplement: Supplementary file 3 — Reporting summary [file 44271_2024_113_MOESM3_ESM.pdf]

nature portfolio

Corresponding author(s): DBPR COMMSPSYCHOL-23-0363

Last updated by author(s): Jun 10, 2024

Reporting Summary

Nature Portfolio wishes to improve the reproducibility of the work that we publish. This form provides structure for consistency and transparency in reporting. For further information on Nature Portfolio policies, see our Editorial Policies and the Editorial Policy Checklist.

Statistics

For all statistical analyses, confirm that the following items are present in the figure legend, table legend, main text, or Methods section.

n/a	Confirmed	
☐	☒	The exact sample size (*n*) for each experimental group/condition, given as a discrete number and unit of measurement
☒	☐	A statement on whether measurements were taken from distinct samples or whether the same sample was measured repeatedly
☐	☒	The statistical test(s) used AND whether they are one- or two-sided<br>*Only common tests should be described solely by name; describe more complex techniques in the Methods section.*
☐	☒	A description of all covariates tested
☐	☒	A description of any assumptions or corrections, such as tests of normality and adjustment for multiple comparisons
☐	☒	A full description of the statistical parameters including central tendency (e.g. means) or other basic estimates (e.g. regression coefficient) AND variation (e.g. standard deviation) or associated estimates of uncertainty (e.g. confidence intervals)
☐	☒	For null hypothesis testing, the test statistic (e.g. *F*, *t*, *r*) with confidence intervals, effect sizes, degrees of freedom and *P* value noted<br>*Give P values as exact values whenever suitable.*
☒	☐	For Bayesian analysis, information on the choice of priors and Markov chain Monte Carlo settings
☒	☐	For hierarchical and complex designs, identification of the appropriate level for tests and full reporting of outcomes
☐	☒	Estimates of effect sizes (e.g. Cohen's *d*, Pearson's *r*), indicating how they were calculated

Our web collection on statistics for biologists contains articles on many of the points above.

Software and code

Policy information about availability of computer code

Data collection	No software was used to collect data.
Data analysis	SPSS version 28/29 for main analyses, Process macro for SPSS version 4.2 for Johnson-Neyman analysis, R version 4.3 (packages tidyverse, sjplot, lme4, lmerTest, cowplot, interactions, sandwich) for replicating main analyses and multi level models, and R Studio Version 2023.06.1 +524 for data visualizations (using ggplot2 and sjplot). Analysis code is available here https://osf.io/8dbyr/?view_only=518991be73624a57b2644b17e56b8ba6

For manuscripts utilizing custom algorithms or software that are central to the research but not yet described in published literature, software must be made available to editors and reviewers. We strongly encourage code deposition in a community repository (e.g. GitHub). See the Nature Portfolio guidelines for submitting code & software for further information.

Data

Policy information about availability of data

All manuscripts must include a data availability statement. This statement should provide the following information, where applicable:

- Accession codes, unique identifiers, or web links for publicly available datasets
- A description of any restrictions on data availability
- For clinical datasets or third party data, please ensure that the statement adheres to our policy

All data is publicly available via the Open Science Framework project page here: https://osf.io/8dbyr/?view_only=518991be73624a57b2644b17e56b8ba6

Human research participants

Policy information about studies involving human research participants and Sex and Gender in Research.

Reporting on sex and gender	We have reported the gender/sex of award winners in our data. In the manuscript, we use the gender labels "women" and "men".
Population characteristics	There are no human research participants. Our data is categorical meta-science data.
Recruitment	Not applicable.
Ethics oversight	School of Psychology Ethics Committee at University of Kent, Canterbury, United Kingdom.

Note that full information on the approval of the study protocol must also be provided in the manuscript.

Field-specific reporting

Please select the one below that is the best fit for your research. If you are not sure, read the appropriate sections before making your selection.

☐ Life sciences ☒ Behavioural & social sciences ☐ Ecological, evolutionary & environmental sciences

For a reference copy of the document with all sections, see nature.com/documents/nr-reporting-summary-flat.pdf

Behavioural & social sciences study design

All studies must disclose on these points even when the disclosure is negative.

Study description	Quantitative
Research sample	We did not recruit human research participants for this study. Instead we gathered meta-science data from professional social and personality psychology society websites.
Sampling strategy	We aimed to include international social and personality psychology societies which had award winners publicly listed on their websites. We decided not to include national societies because we did not identify a comprehensive list of such organizations and because many of them do not have publicly accessible lists of awards presented in English. Nine societies were generated initially through discussion between the first and last author. Following this, we undertook a further search for relevant societies using those listed on the Psychology Organizations and Conferences website (socialpsychology.org). Doing so identified a further four societies for inclusion. Lastly, a general internet search identified an additional four societies mostly focused on personality and cultural psychology. Then we manually collated information on winners of each award, in each year, given by each society. To maximize the number of data points, we decided not to restrict the range of years in which the award was given, but to collect information on awards from all available years up to and including 2021, the last year the data was collected.
Data collection	Data was collected via psychology society websites and entered manually into a data set. There were no human participants.
Timing	Data was collected between June 2020 and December 2021
Data exclusions	The dataset includes 2700 observations. The n varies slightly between tests because we excluded some categories (e.g., “not applicable” category for gender/sex or award level variable) or there was a small number of missing data for year ($n = 4$). All these cases were from a single society (SESP) and listed year as “pre-1977” on the society website. We decided to leave year blank in these instances. All exclusions for each test are stated in the "statistics and reproducibility" section of the manuscript.
Non-participation	Not applicable
Randomization	Not applicable

Reporting for specific materials, systems and methods

We require information from authors about some types of materials, experimental systems and methods used in many studies. Here, indicate whether each material, system or method listed is relevant to your study. If you are not sure if a list item applies to your research, read the appropriate section before selecting a response.

Materials & experimental systems

n/a	Involved in the study
☒	☐ Antibodies
☒	☐ Eukaryotic cell lines
☒	☐ Palaeontology and archaeology
☒	☐ Animals and other organisms
☒	☐ Clinical data
☒	☐ Dual use research of concern

Methods

n/a	Involved in the study
☒	☐ ChIP-seq
☒	☐ Flow cytometry
☒	☐ MRI-based neuroimaging
